# Influence of Left Bundle Branch Block on the Electrocardiographic Changes Induced by Acute Coronary Artery Occlusion of Distinct Location and Duration

**DOI:** 10.3389/fphys.2019.00082

**Published:** 2019-02-12

**Authors:** Esther Jorge, Eduard Solé-González, Gerard Amorós-Figueras, Dabit Arzamendi, Jose M. Guerra, Xavier Millán, Miquel Vives-Borrás, Juan Cinca

**Affiliations:** Department of Cardiology, Hospital de la Santa Creu i Sant Pau, Institute of Biomedical Research IIB Sant Pau, CIBERCV, Universitat Autònoma de Barcelona, Barcelona, Spain

**Keywords:** myocardial ischemia, coronary occlusion, left bundle block, ECG, swine

## Abstract

**Background:** Electrocardiographic (ECG) diagnosis of acute myocardial ischemia is hampered in the presence of left bundle branch block (LBBB).

**Objectives:** We analyzed the influence of location and duration of myocardial ischemia on the ECG changes in pigs with LBBB.

**Methods:** LBBB was acutely induced in 14 closed chest anesthetized pigs by local electrical ablation. Thereafter, episodes of 5 min catheter balloon occlusion followed by 10 min reperfusion of the left anterior descending (LAD), left circumflex (LCX), and right (RCA) coronary arteries were done sequentially in 5 pigs. Additionally, a 3-h occlusion of these arteries was performed separately in the other 9 pigs. A 15-lead ECG including leads V7 to V9 was continuously recorded.

**Results:** Ablation induced LBBB showed QRS widening, loss of r wave in V1, and predominant R waves in V2 to V9. After 5 min of ischemia the occluded artery could be identified in all cases: the LAD by R waves and ST elevation in V1–V3; the LCX by both ST segment elevation in II, III, aVF, V7 to V9 and ST segment depression in V1 to V4; and the RCA by ST depression and new S-waves in all precordial leads. Three hours after coronary occlusion, ST segment changes declined progressively and only the LAD occlusion could be reliably recognized.

**Conclusion:** LBBB did not mask the ECG recognition of the occluded coronary artery during the first 60 min of ischemia, but 3 h later only the LAD occlusion could be reliably identified. ST elevation in leads V7 to V9 is specific of LCX occlusion and it could be useful in the diagnosis of acute myocardial ischemia in the presence of LBBB.

## Introduction

The diagnosis of acute myocardial infarction (AMI) in patients with left bundle branch block (LBBB) is a clinical challenge because this condition may entail a high mortality (Col and Weinberg, 1972; Hindman et al., 1978) and, moreover, a reliable electrocardiographic (ECG) diagnosis is not often possible (Shlipak et al., 1999; Jain et al., 2011). The central feature limiting the diagnostic accuracy of the ECG in patients with AMI and LBBB is the coexistence of QRS and ST segment abnormalities primarily caused by the disturbed cardiac activation. In the absence of concurrent ischemia, the direction of the ST segment displacements (elevation or depression) secondary to LBBB are opposite to the polarity of the corresponding QRS complex (Willems et al., 1985) and a certain degree of proportionality between the magnitude of the ST segment deviation and the amplitude of the QRS complex can be found.

In the presence of acute myocardial ischemia, the newly generated ST segment changes may still maintain the opposite direction to the QRS complex polarity or, at the contrary, both may turn into the same direction. The ECG algorithms commonly used to recognize an AMI in patients with LBBB are mainly based on the relationship between ST segment/QRS polarity (Sgarbossa et al., 1996) and on the proportionality between the ST segment/QRS amplitude (Smith et al., 2012). These algorithms afforded a good diagnostic specificity but a reduced sensitivity (Tabas et al., 2008). However, they do not consider the location of the coronary occlusion or the timing of the evolving ischemic event. It is not well known to which extent the specific ECG patterns induced by a particular occluded artery are modified by the altered activation-repolarization sequences in hearts with LBBB. Likewise, the well-recognized spontaneous attenuation of the ST segment changes during the course of the ischemic process (Janse et al., 1979; Cinca et al., 1980) will predictably influence the ECG criteria for AMI in presence of LBBB, but this needs to be determined.

This study was designed to analyze the influence of the location and duration of acute myocardial ischemia on the ECG changes in hearts with LBBB. We used a closed-chest swine model of LBBB induced acutely by local radiofrequency current ablation of the specialized left conduction bundle.

## Materials and Methods

### Study Population

This study was carried out on anesthetized, closed-chest, domestic swine (Landrace-Large White cross) weighing 40 kg. The animals were premedicated with midazolam (0.6 mg/kg; 5 mg/ml) and ketamine (12 mg/kg; 100 mg/ml) intramuscularly. General anesthesia was induced with intravenous propofol (2–4 mg/kg; 10 mg/ml) and was maintained with a mixture of oxygen and sevoflurane inhalation (2.5%–3.5%). After endotracheal intubation, the animals were mechanically ventilated. Fentanyl (2.5 μg/kg; 0.05 mg/ml) was administered intravenously during the procedure for maintained analgesia. This study was carried out in accordance with the recommendations of the Guide for the Care and Use of Laboratory Animals, 8th ed. (National Research Council. Washington, DC: The National Academies Press, 2010). The protocol was approved by the Animal Ethics Committee of our Institution.

### Experimental Procedures

#### Radiofrequency (RF) Ablation of the Left Bundle Branch

**FIGURE 1:**
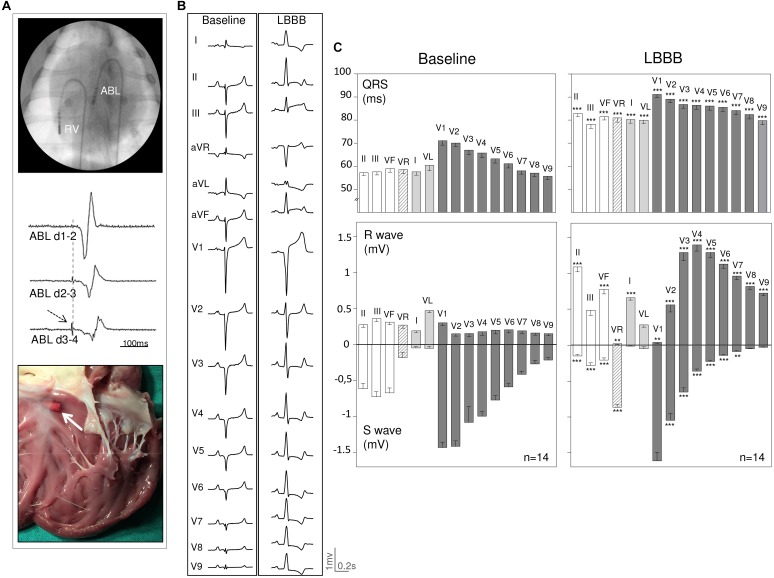
Graphic illustration of the experimental model of left bundle branch block induced in 14 open chest anesthetized pigs. **(A)** From top to bottom shows (1) an angiographic frame depicting the position of the ablation electrocatheter (ABL) in the LV outflow tract close to the region of the left bundle branch region and the pacing electrocatheter in the apex of the right ventricle (RV), (2) endocardial bipolar electrograms from the ABL electrocatheter showing the left bundle potential (arrow), and (3) a macroscopic view of the subaortic LV endocardium showing the ablation lesion (white arrow) close to the left conduction system. **(B)** ECG recording in a pig before and after induction of LBBB. **(C)** Bars illustrate the mean values and whiskers the SEM of the QRS complex duration and R-S wave amplitude before and after induction of LBBB measured in all ECG leads in 14 pigs. ^∗∗^ *p* value < 0.01, ^∗∗∗^ *p* value < 0.001 LBBB vs. baseline. LBBB = Left bundle branch block.

Closed-chest blockade of the left bundle branch was performed following a previously reported model in swine (Rigol et al., 2013). We catheterized the femoral artery and the femoral vein using the Seldinger’s technique. Under fluoroscopic guidance, an ablation electrocatheter (7F Blazer, Boston Scientific^®^) was advanced to the left ventricle (LV) and then positioned just below the aortic valve. A second electrocatheter was placed at the apex of the right ventricle (RV) for pacing purposes. The LV electrocatheter was connected to a Prucka System (Prucka Engineering, Inc., Houston, TX, United States) and was slightly moved in the septal region of the outflow tract to record the left bundle branch potential which was recognized as a sharp spike preceding (<35 ms) the onset of the endocardial ventricular potential (Figure 1A). The right ventricular electrocatheter was connected to a temporary external pacemaker (Medtronic Inc., Minneapolis, MN, United States) and was programmed for regular ventricular pacing (V00) at 160 bpm. RF applications (30 W, 30 s) were delivered in the region of the left bundle potential until a LBBB pattern was recorded in the ECG. Since pigs are prone to develop ventricular fibrillation under application of RF current, we administered a continuous intravenous perfusion of amiodarone (300 mg/h) and immediately before the application of RF ablation, we paced the RV at 160 bpm to homogenize the ventricular refractoriness. Once the LBBB was created, the RV electrocatheter was placed to the lateral wall of the right atrium to pace the heart at 105 bpm throughout the study. This procedure was done to permit a better comparison of the ECG changes induced among the study pigs. The LBBB pattern was defined by the appearance of: (1) widening of QRS complex associated with secondary ST segment and T wave changes; (2) QS pattern in lead V1; and (3) switch from the baseline r/S complex pattern in left precordial leads to a predominant R wave pattern with absence of Q waves.

#### Coronary Artery Occlusion

Acute transmural myocardial ischemia was induced by percutaneous coronary catheter balloon occlusion. We introduced a 6F guiding catheter (Cordis) into a femoral artery and it was advanced to the ostium of the left or the right coronary arteries under fluoroscopic guidance Then, a 3 mm diameter over-the-wire catheter balloon (Cordis) was placed at the mid segment of one of the three main coronary arteries. The appropriate position of the balloon was confirmed by coronary angiography. Anticoagulation was induced in all cases by an intravenous bolus of unfractionated heparin (150 UI/kg) followed by sequential boluses (100 UI/kg) hourly until the end of the study.

#### 15 Lead-Electrocardiogram (ECG)

A 15 lead-ECG was continuously recorded and stored on a CardioSoft ECG system (Version 6.7.3, GE Healthcare, Freiburg, Germany). Due to the species anatomy, we placed the conventional precordial leads V1-V6 one intercostal space above that used in current clinical electrocardiography (Noriega et al., 2013). The three additional posterior chest leads V7–V9 were placed after lead V6 at regular interelectrode spaces. The ECG was recorded at baseline, after induction of LBBB, and continuously during the coronary occlusion sequences. In each ECG recording, we measured the following parameters using electronic calipers (Cardio-Calipers software, Iconico©) and imaging magnification: (1) QRS complex duration; (2) amplitude of the R wave and S wave; and (3) ST segment displacement at the J point.

### Study Protocol

To achieve the main objectives of the study we delineated two experimental protocols.

#### Protocol #1

Attempted to compare the ECG changes induced by ischemia in three different myocardial regions in the same animal. Each case was sequentially submitted to a 5-min occlusion followed by 10 min of reperfusion of the mid segments of the left anterior descending (LAD), left circumflex (LCX), and right coronary (RC) arteries. In all instances the ischemic ECG changes recovered during the reperfusion period. To minimize the preconditioning effect of repeated occlusions in the same heart, we randomized the occlusion sequences among the study pigs.

#### Protocol #2

Addressed to describe the effect of time on the ECG changes along 3 hours of coronary occlusion analyzed separately in three different myocardial territories in pigs with LBBB. The animals were divided into three groups according to the occluded coronary artery: LAD, LCX, or RCA. In all cases the ECG was continuously recorded during the coronary occlusion sequences.

At the end of the study the animals were euthanized with intravenous injection of potassium chloride.

### Data Analysis

Quantitative data were expressed as the mean ± standard error of the mean (SEM). The changes in the ECG variables from baseline to LBBB induction were evaluated by the analysis of variance (ANOVA) with Bonferroni correction for *post hoc* comparisons. A *p* value < 0.05 was considered significant. All analysis were performed using SPSS v.22.0 (IBM SPSS Inc., Chicago, IL, United States).

## Results

### Study Population

An effective block of the left bundle was achieved in 22 out of 29 pigs (76% rate of success). The 22 pigs with LBBB were submitted to the two study protocols and 8 of them (4 in each protocol) died because of ventricular arrhythmias or AV block induced during the coronary occlusion sequences. Therefore, 14 pigs with LBBB completed the entire study (5 pigs in protocol #1 and 9 in protocol #2). All animals included in the study were free of significant atherosclerotic coronary artery disease.

### LBBB Pattern Induced by RF Ablation

As illustrated in Figure 1A,B, application of RF current in the endocardial sites with recorded local left bundle branch potentials, induced a LBBB pattern characterized by: (a) widening of the QRS complex associated with secondary ST segment changes, (b) disappearance of the r wave in lead V1, (c) abrupt appearance of predominant R waves in leads V2 to V9 contrasting with the pre-ablation rS complex seen in these leads, and (d) change in the QRS axis from a left deviation at baseline to an AQRS of about 60°. The quantitative changes in QRS complex duration and R/S wave amplitude elicited by left bundle RF ablation in the 14 included pigs with LBBB pigs are illustrated in Figure 1C.

### Effects of Location of Coronary Occlusion on the ECG Changes (Protocol #1)

**FIGURE 2:**
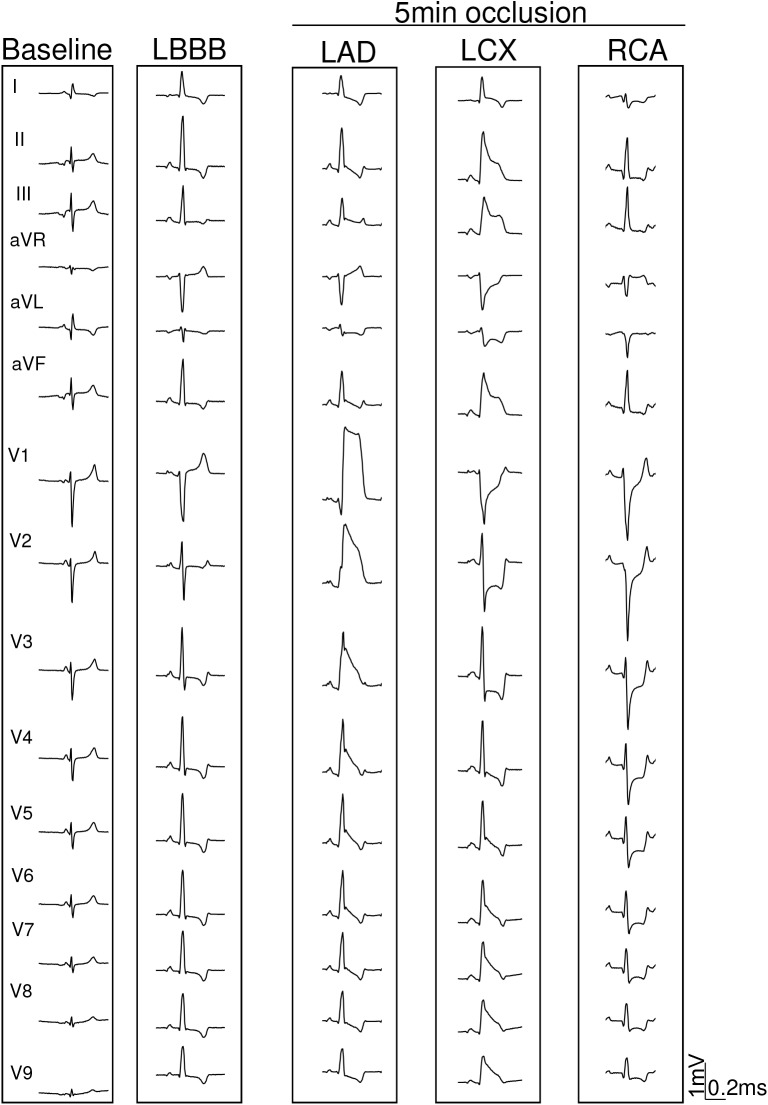
Representative ECG after 5 min of myocardial ischemia in one pig with left bundle branch block (LBBB) induced by electrical ablation. ECG changes induced by 5 min occlusion of the left anterior descending (LAD), left circumflex (LCX), and right (RCA) coronary arteries in the same pig.

**FIGURE 3:**
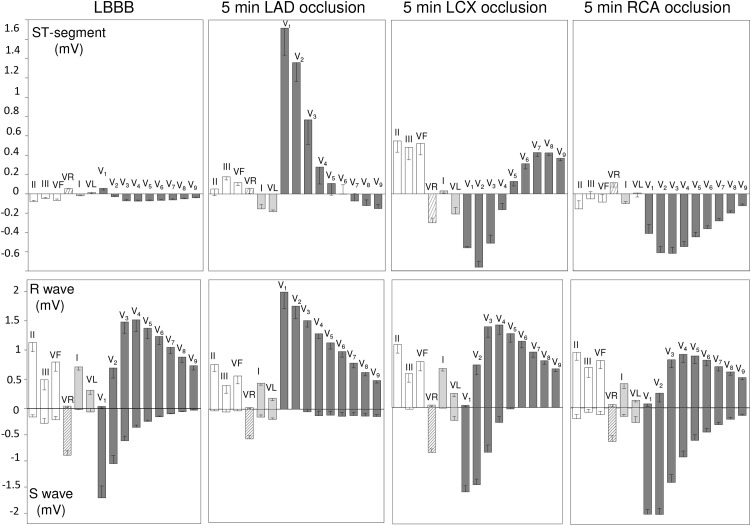
ECG changes induced by 5 min coronary occlusion in 5 pigs with left bundle branch block (LBBB) induced by electrical ablation. Bars represent the mean values and whiskers the SEM of the ST segment (upper panels), and R- S waves (lower panels) LBBB and after 5 min occlusion of the left anterior descending (LAD), left circumflex (LCX), and right (RCA) coronary arteries.

Series of 5 min occlusion–10 min reperfusion involving sequentially the LAD, LCX, and RCA of the same subject were performed in 5 pigs with LBBB. Typical ECG tracings recorded during the separate occlusion of the 3 coronary arteries are presented in Figure 2 and the mean ECG changes for the entire group are graphically represented in Figure 3. Occlusion of the LAD induced: (a) Increased R waves with ST segment elevation (monophasic potentials) in leads V1 to V3; and (b) slight reciprocal ST segment depression in leads V7-V9. Occlusion of the LCX induced ST segment elevation in leads II, III, aVF, and V6 to V9 with reciprocal ST segment depression in leads V1 to V3. Occlusion of the RCA elicited ST segment depression in all precordial leads (from V1 to V9) with decreased R wave voltage and reappearance of S waves in these leads. In our model we did not find appreciable ST segment elevation in leads II, III, or aVF.

### Effects of Duration of Coronary Occlusion on the ECG Changes (Protocol #2)

**FIGURE 4:**
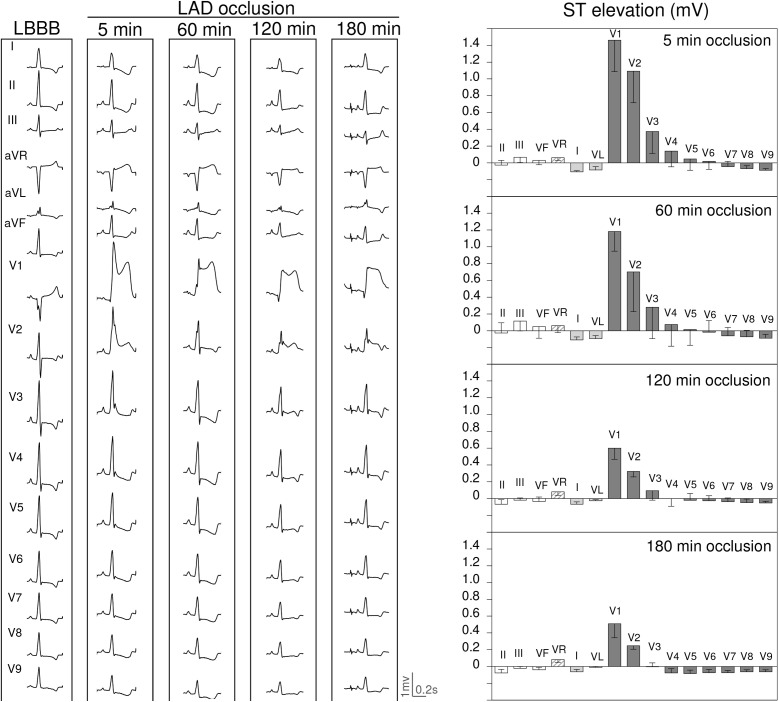
ECG changes induced by 180 min occlusion of the left anterior descending (LAD) coronary artery in 3 pigs with left bundle branch block (LBBB) induced by electrical ablation. Left panel: ECG recordings at 5, 60, 120, and 180 min of LAD occlusion in a pig with LBBB. Right panel: Bars illustrate the mean values and whiskers the SEM of the ST segment deviation at 5, 60, 120, and 180 min of LAD occlusion in 3 pigs with LBBB.

**FIGURE 5:**
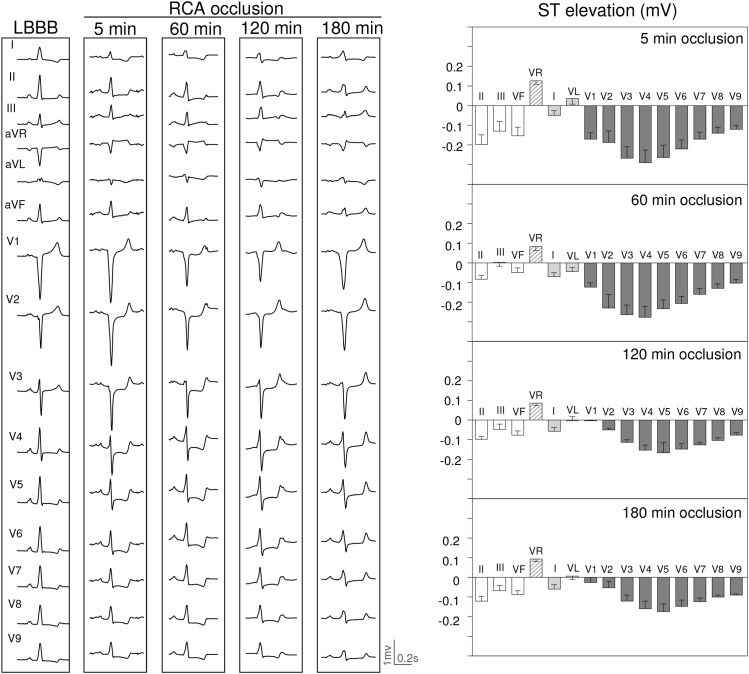
ECG changes induced by 180 min occlusion of the right (RCA) coronary artery in 3 pigs with left bundle branch block (LBBB) induced by electrical ablation. Left panel: ECG recordings at 5, 60, 120, and 180 min of RCA occlusion in a pig with LBBB. Right panel: Bars illustrate the mean values and whiskers the SEM of the ST segment deviation at 5, 60, 120, and 180 min of RCA occlusion in 3 pigs with LBBB.

**FIGURE 6:**
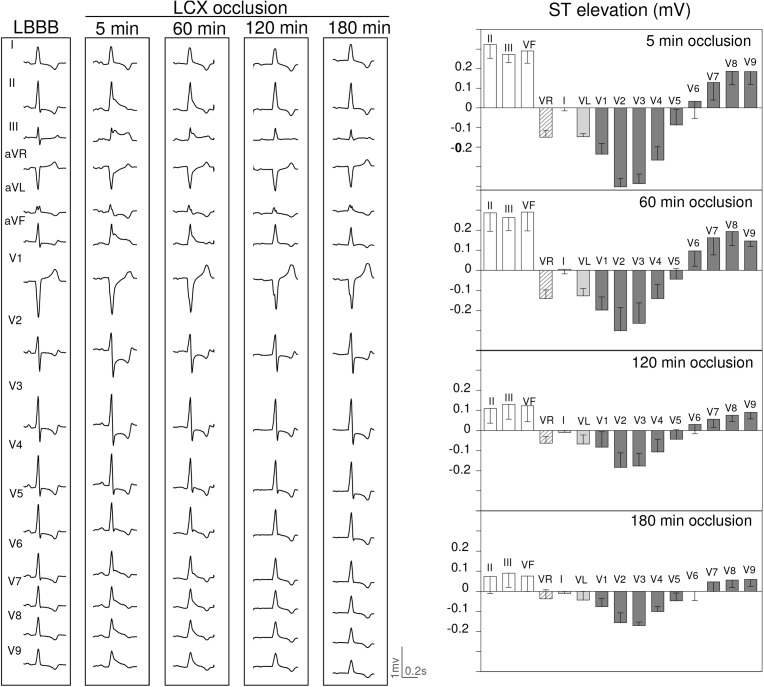
ECG changes induced by 180 min occlusion of the left circumflex (LCX) coronary artery in 3 pigs with left bundle branch block (LBBB) induced by electrical ablation. Left panel: ECG recordings at 5, 60, 120, and 180 min of LCX occlusion in a pig with LBBB. Right panel: Bars illustrate the mean values and whiskers the SEM of the ST segment deviation at 5, 60, 120, and 180 min of LCX occlusion in 3 pigs with LBBB.

Figure 4–6 summarize the time course of the ischemic ECG changes during 180 min of coronary occlusion. As compared with the first 5 min of ischemia, the magnitude of the ST segment changes decreased progressively over time in the three explored coronary territories. However, 180 min after coronary occlusion the ST segment deviation still remained apparent in pigs with LAD occlusion (Figure 4), but underwent mostly unappreciable in cases with RCA (Figure 5) and especially in those with LCX occlusion (Figure 6). The magnitude of the R wave changes in leads V1–V2 in cases of LAD occlusion also decreased over time. The trends of the R/S patterns in precordial leads remained comparable after 180 min of ischemia in pigs with LCX and RCA occlusion. To comparatively analyze the ECG changes in the three coronary territories at the same scale, Supplementary Figure S1 illustrates the quantitative ST segment changes and Supplementary Figure S2 typical 15-lead ECG patterns.

## Discussion

### Main Findings

In this study we have analyzed systematically the influence of the location and time duration of acute myocardial ischemia on the ECG changes in pigs with LBBB. Our data revealed that within the first 5 min of ischemia, the ECG changes could identify the LAD, LCX and RCA occlusion. However, 3 h later, only involvement of the LAD was reliably recognizable. In addition, our study highlighted the utility of the posterior thoracic leads V7 to V9 to differentiate occlusion of the LCX in the presence of LBBB.

### Effects of Location and Duration of Acute Myocardial Ischemia

Electrocardiographic diagnosis of acute coronary occlusion in patients presenting a concomitant LBBB is frequently uncertain. Indeed, studies in cases with acute chest pain and new or presumably new LBBB submitted to primary percutaneous coronary intervention revealed that only 39% of cases had the final diagnosis of AMI (Jain et al., 2011). To improve the diagnostic accuracy of the ECG, several algorithms have been proposed but often they only afford a modest sensitivity and, on the other hand, were not purposely designed to consider the location or the duration of the coronary occlusion (Sgarbossa et al., 1996; Smith et al., 2012).

In the absence of intraventricular conduction defects, the site of coronary occlusion is well recognized by the widely established ECG criteria (Wagner et al., 2009; Noriega et al., 2014), but in the presence of LBBB the validity of these ECG patterns has not been fully confirmed. The delayed and heterogeneous activation of the LV in hearts with LBBB alters the normal cardiac repolarization sequence and consequently, the mismatch between the spatial location of the acute ischemic region and the moment of its local repolarization would likely influence the ensuing ischemic changes in QRS morphology and ST segment displacement.

Our study affords novel data on the potential influence (i.e., “masking effect”) of the LBBB on the ECG patterns induced by separate acute occlusion of the LAD, LCX, and RCA in the same individual. Taking as a reference a previous study in pigs not submitted to left bundle ablation (Vives-Borrás et al., 2018), the present data showed that in the very early stages of coronary occlusion the affected coronary segment depicts similar ECG patterns both in pigs with and without LBBB.

A constant characteristic of a permanent occlusion of a coronary artery is that the magnitude of the ischemic ST segment changes decline spontaneously over time due to a concurrent increase of the electrical tissue resistivity inside the ischemic myocardial region (Kléber et al., 1987; Cinca et al., 1997). The progressive attenuation of the ST segment changes after 3 h of coronary occlusion was noticeable in our cases. At this stage only the LAD occlusion could be reliably recognized since the ST segment changes in cases of LCX and RCA occlusion mostly returned to baseline levels within 3 h.

### Study Limitations

The reliability of the swine model of LBBB used in this study is based on: (a) the abrupt increase in QRS complex duration and appearance of R waves with secondary ST segment changes in precordial leads, and (b) the development of an abnormal LV contraction pattern comparable to that observed in patients with LBBB as was described in a similar swine model (Rigol et al., 2013). Moreover, the characteristic delay in the interventricular septal activation found in patients with LBBB has been also confirmed by LV electroanatomical mapping recordings in pigs with ablation induced LBBB (Soto Iglesias et al., 2017).

The QRS duration both at baseline and after induction of LBBB in pigs is shorter than that observed in patients with and without LBBB. This species difference would likely relay on the distinct transmural distribution of their specialized conduction system. Thus, a more transmural penetration of Purkinje distribution in pigs (Holland and Brooks, 1976) allows a faster ventricular excitation and a rather simultaneous epicardial and endocardial activation (Hamlin et al., 1975; Ideker and Huang, 2005) as compared with a wider spread of the endocardial to epicardial activation in men (Durrer et al., 1970; Opthof et al., 2017).

In addition, the absence of progressively increasing R waves from lead V1 to V6 is a species characteristic of pigs could be in part due to the vertical intrathoracic position of their heart. This particular intrathoracic position can also explain that acute ischemia in the LV inferior region secondary to acute RCA occlusion could evoke modest ST segment elevation in leads II, III, and aVF in pigs, as compared with humans. However, in both species the reciprocal ST segment depression induced by occlusion of the RCA was remarkable in precordial leads V1 to V4.

Even though we cannot entirely exclude a preconditioning effect on the ST segment changes after repeated coronary occlusions, we have observed noticeable ST segment changes during the first five minutes of occlusion in each of the three coronary arteries explored. Moreover, the objective of the study was not establishing a quantitative comparison of the ST segment changes among the three ischemic regions. To minimize the preconditioning effect of repeated occlusions in the same heart, we randomized the occlusion sequences among the study pigs.

Although the incidence of multivessel disease in patients with acute STEMI and LBBB is relatively high, clinical extrapolation of our results may be supported by the fact that in a previous study (Noriega et al., 2014) we found that patients with STEMI with single or multivessel coronary disease have concordant artery-related ST-segment patterns.

### Clinical Implications

Extrapolation of the electrocardiographic findings of our study to medical practice is tenable because the coronary distribution and cellular electrophysiologic derangements caused by myocardial ischemia in pigs are similar to humans (Cinca et al., 1980), as are the ECG patterns induced by selective occlusion of the LAD, LCX, or RCA in both species (Noriega et al., 2014; Vives-Borrás et al., 2018). Consequently, the present data suggest that during the first 60 min of a coronary occlusion, the location of the culprit vessel in patients with LBBB could be predicted by the classical electrocardiographic patterns described in patients free of conduction disturbances. By contrast, 3 h later only the occlusion of the LAD would remain identifiable by the ECG.

A matter of debate in patients with ST segment elevation myocardial infarction (STEMI) and LBBB is whether the clinical management should be approached differently if the conduction block had appeared acutely in the context of the AMI, or anteceded the ischemic event. The current guidelines recommend a similar emergent reperfusion referral policy independently of the time of presentation of the LBBB (Ibanez et al., 2018). Our data will support this recommendation but mainly in patients attended 60 min beyond the onset of chest pain because in earlier stages of ischemia, the masking effect of a preexisting LBBB on the ECG changes might be negligible. Furthermore, if the LBBB had appeared acutely in the course of the ischemic process, then the masking influence on the ECG changes would be even less notorious because the ischemic LBBB is often associated with large infarcts and these are expected to induce overt acute ST segment and QRS changes.

A practical recommendation derived from our study is the pertinence to routinely record the posterior thoracic leads V7 to V9 in patients with acute coronary syndromes based on the high diagnostic yielding of these leads in cases of LCX occlusion. In a series of patients with LCX occlusion (Vives-Borrás et al., 2017) we have found that leads V7 to V9 depict ST segment elevation even in cases showing only a ST segment depression pattern in the whole ECG. In the swine model we have also confirmed that in contrast to LAD or RCA, the LCX occlusion was the only one depicting ST segment elevation in leads V6 to V9 (Vives-Borrás et al., 2018). The present study extends this observation to pigs with LBBB, thus giving foundation to further clinical studies analyzing the validity of this sign to diagnose LCX occlusion in patients with LBBB.

## Conclusion

By handling a swine model of ablation induced LBBB our data support the hypothesis that in patients attended during the first 60 min of acute coronary occlusion, the presence of a preexisiting LBBB will not impede to diagnose an ischemic event or to localize the culprit coronary artery guided by the ECG changes. By contrast, 3 h later, only occlusion of the LAD will be identifiable. Prospective clinical studies considering the location and duration of the ischemia are needed to confirm the present hypothesis aimed at improving the guideline’s recommendations.

## Author Contributions

JC conceived and design of the research. EJ, ES-G, GA-F, DA, JG, XM, MV-B, and JC performed the experiments, edited and revised manuscript, and approved the final version of the manuscript. EJ, ES-G, GA-F, and JC analyzed data and interpreted results of experiments, prepared the figures and drafted the manuscript.

## Conflict of Interest Statement

The authors declare that the research was conducted in the absence of any commercial or financial relationships that could be construed as a potential conflict of interest.
