# Supplementary material for: Influence of Left Bundle Branch Block on the Electrocardiographic Changes Induced by Acute Coronary Artery Occlusion of Distinct Location and Duration
Source: Front Physiol. 2019 Feb 12;10:82. doi: 10.3389/fphys.2019.00082 (PMC6379473; doi:10.3389/fphys.2019.00082)
Supplement: Supplementary file 1 [file Data_Sheet_1.pdf]

Supplementary Material

1 Supplementary Figures

Supplementary Figure 1

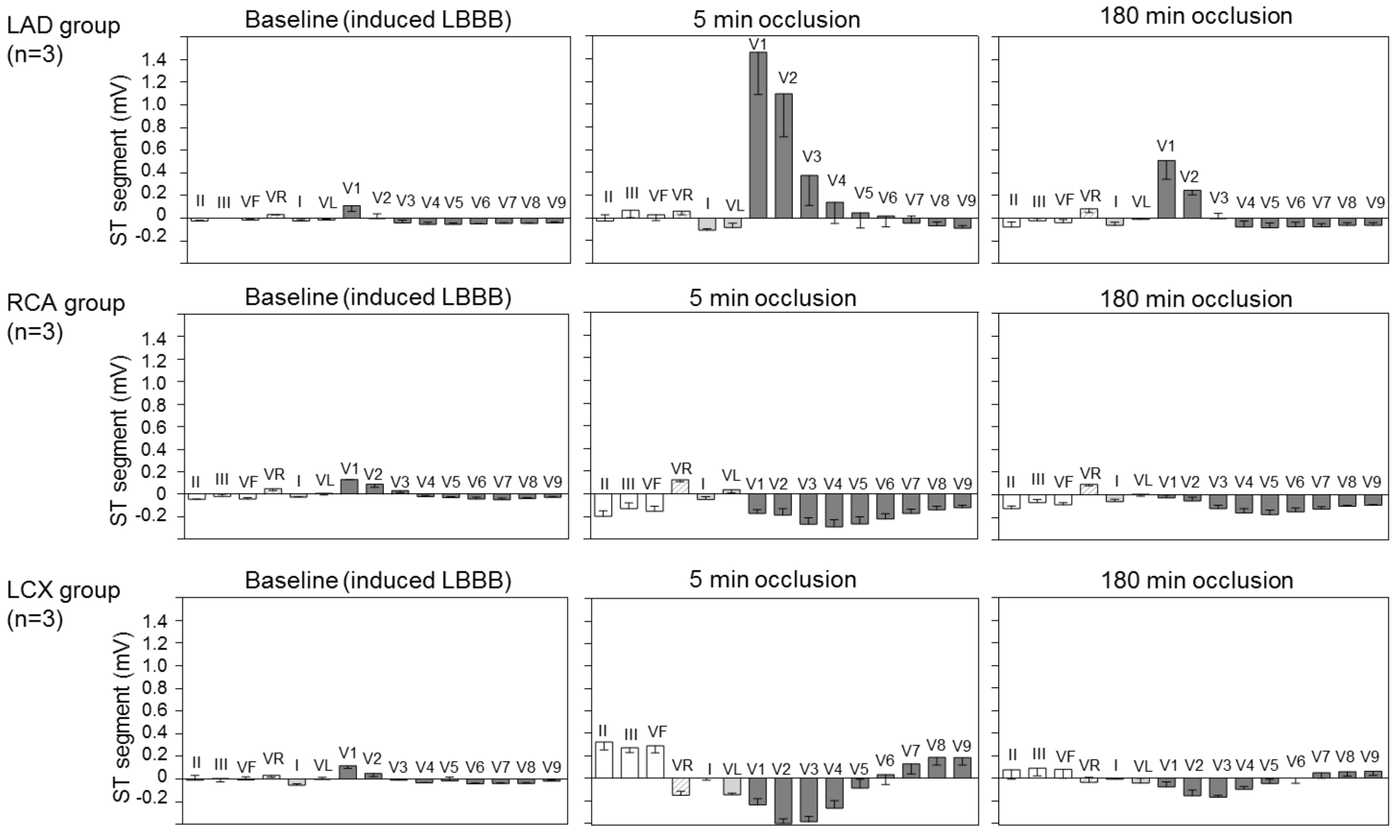

Supplementary Figure 1. ST segment changes induced by acute coronary artery occlusion in three different coronary regions in pigs with electrical ablation induced left bundle branch block (LBBB). Bars illustrate the mean values and whiskers the SEM of the ST segment deviation after LBBB induction and after 5 and 180 min of left anterior descending (LAD), left circumflex (LCX), and right (RCA) coronary arteries.

Supplementary Figure 2

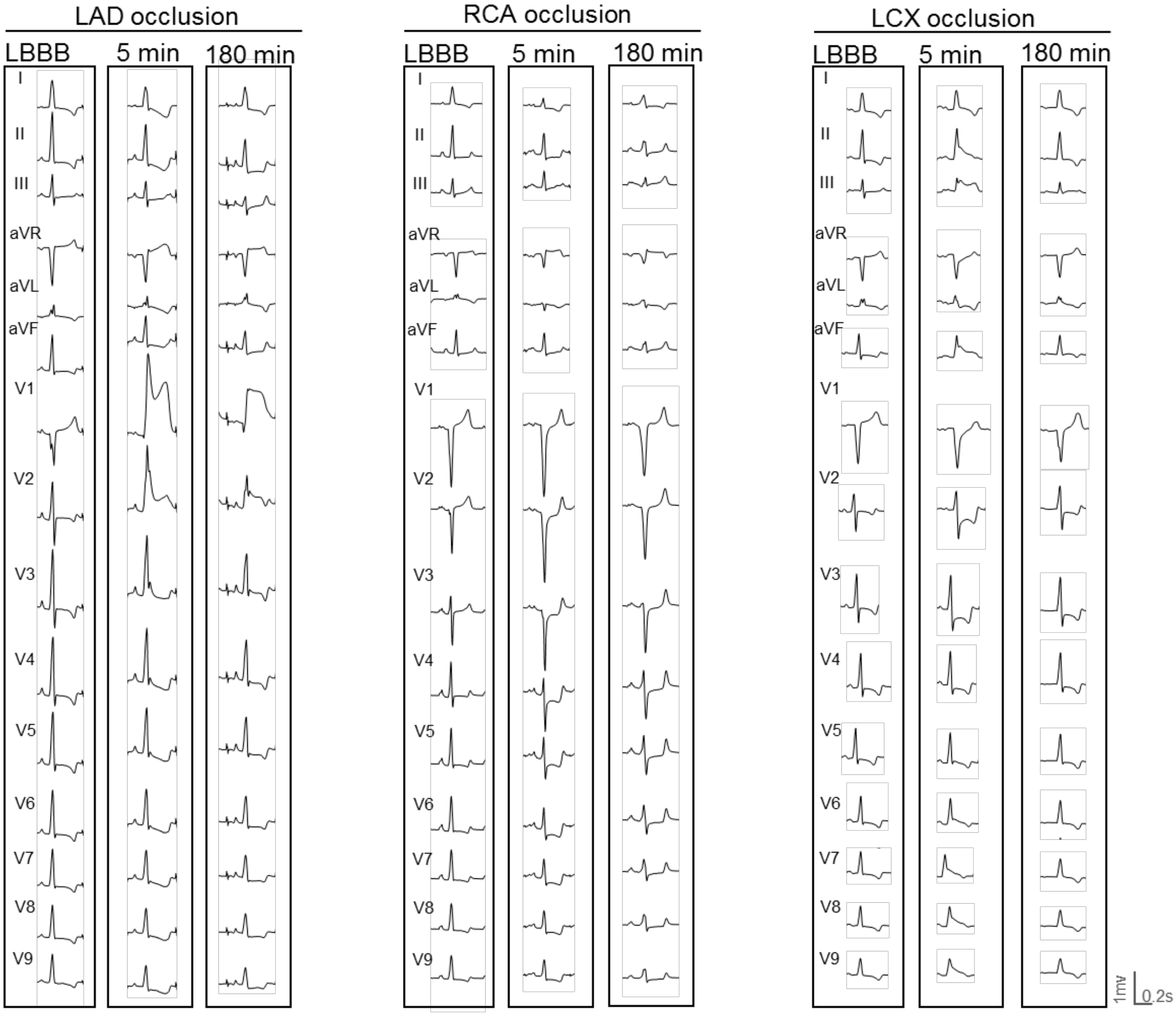

Supplementary Figure 2. 15-lead ECG changes after 5, and 180 min of acute occlusion of the three coronary arteries in pigs with electrical ablation induced left bundle branch block. Abbreviations: LAD=Left anterior descending coronary artery, RCA=Right coronary artery, LCX=Left circumflex artery.
